# Artemether-Lumefantrine versus Dihydroartemisinin-Piperaquine for Treatment of Malaria: A Randomized Trial

**DOI:** 10.1371/journal.pctr.0020020

**Published:** 2007-05-18

**Authors:** Moses R Kamya, Adoke Yeka, Hasifa Bukirwa, Myers Lugemwa, John B Rwakimari, Sarah G Staedke, Ambrose O Talisuna, Bryan Greenhouse, François Nosten, Philip J Rosenthal, Fred Wabwire-Mangen, Grant Dorsey

**Affiliations:** 1 Department of Medicine, Makerere University, Kampala, Uganda; 2 Uganda Malaria Surveillance Project, Kampala, Uganda; 3 Uganda Ministry of Health, Kampala, Uganda; 4 London School of Hygiene and Tropical Medicine, London, United Kingdom; 5 Department of Medicine, University of California San Francisco, San Francisco, California, United States of America; 6 Faculty of Tropical Medicine, Mahidol University, Bangkok, Thailand; 7 Institute of Public Health, Makerere University, Kampala, Uganda

## Abstract

**Objectives:**

To compare the efficacy and safety of artemether-lumefantrine (AL) and dihydroartemisinin-piperaquine (DP) for treating uncomplicated falciparum malaria in Uganda.

**Design:**

Randomized single-blinded clinical trial.

**Setting:**

Apac, Uganda, an area of very high malaria transmission intensity.

**Participants:**

Children aged 6 mo to 10 y with uncomplicated falciparum malaria.

**Intervention:**

Treatment of malaria with AL or DP, each following standard 3-d dosing regimens.

**Outcome measures:**

Risks of recurrent parasitemia at 28 and 42 d, unadjusted and adjusted by genotyping to distinguish recrudescences and new infections.

**Results:**

Of 421 enrolled participants, 417 (99%) completed follow-up. The unadjusted risk of recurrent falciparum parasitemia was significantly lower for participants treated with DP than for those treated with AL after 28 d (11% versus 29%; risk difference [RD] 18%, 95% confidence interval [CI] 11%–26%) and 42 d (43% versus 53%; RD 9.6%, 95% CI 0%–19%) of follow-up. Similarly, the risk of recurrent parasitemia due to possible recrudescence (adjusted by genotyping) was significantly lower for participants treated with DP than for those treated with AL after 28 d (1.9% versus 8.9%; RD 7.0%, 95% CI 2.5%–12%) and 42 d (6.9% versus 16%; RD 9.5%, 95% CI 2.8%–16%). Patients treated with DP had a lower risk of recurrent parasitemia due to non-falciparum species, development of gametocytemia, and higher mean increase in hemoglobin compared to patients treated with AL. Both drugs were well tolerated; serious adverse events were uncommon and unrelated to study drugs.

**Conclusion:**

DP was superior to AL for reducing the risk of recurrent parasitemia and gametocytemia, and provided improved hemoglobin recovery. DP thus appears to be a good alternative to AL as first-line treatment of uncomplicated malaria in Uganda. To maximize the benefit of artemisinin-based combination therapy in Africa, treatment should be integrated with aggressive strategies to reduce malaria transmission intensity.

## Editorial Commentary

**Background:** The burden of death and disease caused by malaria is very high, particularly amongst young children in sub-Saharan Africa. Many African countries have adopted combinations of drugs for first-line malaria treatment that include a compound based on the plant-derived molecule artemisinin. In Uganda, the artemisinin-based compound therapy (ACT) adopted as first-line therapy is artemether-lumefantrine (AL). However, there are limitations to the use of this drug; dosing is not convenient and it needs to be given with fatty food. There are also concerns that even though AL and other ACTs successfully treat the initial infection, there is a risk of malaria recurring soon after therapy, particularly in areas where mosquito attack rates are high. Therefore, the researchers here wanted to compare AL treatment with another ACT, dihydroartemisinin-piperaquine (DP), for treatment of uncomplicated malaria in children who live in an area of Uganda where malaria is transmitted at a very high rate.

**What the trial shows:** 421 children aged between six months and ten years arriving at a local health centre with uncomplicated malaria were recruited into the study. The children were randomly assigned to receive either AL tablets, given twice daily for 3 days, or DP tablets, given once daily for 3 days. The primary outcomes in the trial were the risks of clinical malaria recurring, and the risk of parasites reappearing in the blood but without any sign of clinical malaria, over 42 days of follow-up. The researchers also carried out tests to distinguish new malaria infections from a reappearance of the old infection.

Overall, children who received DP treatment were less likely to have malaria parasites reappear in their blood than children who received AL treatment. Similarly, the risk of a recurrence of clinical malaria was lower in the DP arm than in the AL arm; 39% of patients treated with AL went on to develop malaria symptoms by 42 days whilst only 25% of patients treated with DP did. These findings were similar if the researchers only looked at children in whom the same infection reappeared, as opposed to those who experienced a completely new infection. There did not seem to be any differences between the study groups in overall rates of adverse events, most of which were mild or moderate in nature. The serious adverse events that occurred in this trial were not thought to be related to the drugs being studied.

**Strengths and limitations:** This trial recruited enough participants to test whether there were any differences in efficacy between the two treatments studied, and randomization procedures and blinding of study physicians were appropriate. A further strength is the follow-up period of 42 days, in line with recent World Health Organization guidelines for such trials. This trial included children aged under 10 years, whereas children aged under 5 years are generally considered most at risk from malaria. However, the majority of children in this trial were in the under-5 age group, and the overall results were similar when only considering children in this age range. Finally, one possible limitation is that blinding of participants may not have been complete in this trial: children and parents were not informed of their treatment assignment, but the study drugs were different in appearance.

**Contribution to the evidence:** Studies from South-East Asia have indicated that DP has efficacy in treating malaria resistant to other drugs. However, little data exists regarding the efficacy and safety of DP in Africa. This trial adds important safety data for DP in African children, and also provides evidence that DP could be considered as an alternative treatment to AL, the current first-line therapy in many African countries.

## INTRODUCTION

In Africa, treatment of uncomplicated malaria is undergoing dramatic changes. In response to widespread resistance of the parasite to commonly used monotherapies, particularly chloroquine (CQ) and sulfadoxine-pyrimethamine (SP), many countries have recently adopted artemisinin-based combination therapy (ACT) as a first-line regimen for the treatment of uncomplicated malaria [1]. In Uganda, artemether-lumefantrine (AL) was chosen to replace the combination of CQ + SP as the first-line regimen for malaria in 2004, with amodiaquine + artesunate (AQ + AS) offered as an alternative regimen. Although data on AL were limited at that time, subsequent studies from Uganda [2,3] and elsewhere in Africa [4–6] showed that AL was highly efficacious and well tolerated for uncomplicated malaria. Indeed, in the two published studies of AL from Uganda the risk of treatment failure due to recrudescence was reported to be less than 2% [2,3]. However, despite the promise of AL, there are substantial limitations to this regimen, including twice-daily dosing and need for administration with fatty food.

A concern with all antimalarial regimens, particularly in areas of high transmission, is frequent recurrence of malaria after therapy. This concern was highlighted in a recent study comparing efficacies of the ACTs AL and AQ + AS in Tororo, Uganda, an area with very high malaria transmission [3]. In that study, both regimens were highly efficacious for eradication of infections but risks of reinfection were extremely high, with 50% of AL-treated and 66% of AQ + AS–treated patients developing recurrent parasitemia within 28 d.

Dihydroartemisinin-piperaquine (DP) is a fixed-dose ACT that has recently become available in Africa. In studies from Southeast Asia, DP appeared to be well tolerated and highly efficacious against multidrug-resistant falciparum malaria [7–11]. However, the epidemiology of malaria and patterns of antimalarial drug use are quite different in Africa than in Asia [12,13]. In the only published study evaluating DP in Africa, DP was highly efficacious with a good safety and tolerability profile at three sites in Rwanda [14]. To compare the performance of DP with the new first-line therapy in Uganda, we conducted a randomized clinical trial comparing the efficacy and safety of AL and DP for the treatment of uncomplicated falciparum malaria in Apac, an area of extremely high transmission intensity.

## METHODS

### Study Site

The study was conducted at Aduku Health Centre, Apac District, Uganda. The district experiences perennial holoendemic malaria. The entomological inoculation rate in Apac, a measure of transmission intensity, was measured at 1,564 infectious bites per person per year [15]. The study protocol was approved by the Makerere University Research and Ethics Committee, the Uganda National Council of Science and Technology, and the University of California San Francisco Committee for Human Research.

### Participants

Consecutive patients presenting to the health center with symptoms suggestive of malaria and a positive screening thick blood smear were referred to study physicians for further assessment. Patients were enrolled if they fulfilled the following selection criteria: (1) age 6 mo to 10 y; (2) weight ≥ 5 kg; (3) history of fever in the last 24 h or axillary temperature ≥ 37.5 °C; (4) no history of serious side effects to study medications; (5) no evidence of a concomitant febrile illness; (6) provision of informed consent by a parent or guardian; (7) no danger signs or evidence of severe malaria; and (8) Plasmodium falciparum monoinfection with parasite density 2,000–200,000/μl of blood. Because laboratory results were generally not available until the following day, a patient could be excluded after randomization.

### Procedures

At enrollment, we asked children's parents or guardians about prior antimalarial therapy, use of other medications, and presence of common symptoms. Axillary temperature and weight were measured, and a physical examination was performed. A brief neurological assessment, consisting of simple clinical tests for fine finger dexterity (ability to pick up a small object), was undertaken. We also obtained blood by fingerprick for thick and thin blood smears, for hemoglobin assessment, and to store on filter paper for molecular analysis.

Patients were asked to return for follow-up on days 1, 2, 3, 7, 14, 21, 28, 35, and 42, and any other day that they felt ill. Follow-up evaluation consisted of a standardized history and physical examination, including neurological assessment on all days of follow-up. We obtained blood by fingerprick for thick blood smears and storage on filter paper on all follow-up days except day 1. Hemoglobin measurement was repeated on day 42 or the day of recurrent symptomatic malaria. If patients did not return for follow-up, they were visited at home.

Blood smears were stained with 2% Giemsa for 30 min. Parasite densities were determined from thick blood smears by counting the number of asexual parasites per 200 white blood cells (WBCs), or per 500 if the count was less than 10 parasites/200 WBCs, assuming a WBC count of 8,000/μl. A smear was considered negative if no parasites were seen after review of 100 high-power fields. We also assessed gametocytemia from thick blood smears. Thin blood smears were reviewed for non-falciparum infections. A second microscopist, who was unaware of the results of the first reading, re-read all slides. A third microscopist unaware of the first two readings resolved discrepant slides. Hemoglobin measurements were made using a portable spectrophotometer (HemoCue, http://www.hemocue.com).

### Interventions

On day 0, patients were randomly assigned to receive AL or DP. A nurse administered study medications according to weight-based guidelines for administration of fractions of tablets. We administered all drugs orally as follows: AL (Coartem, Novartis, 20 mg artemether/120 mg lumefantrine tablets), administered according to weight as one (5–14 kg), two (15–24 kg), three (25–34 kg), or four (≥ 35 kg) tablets given twice daily for 3 d; DP (Duocotexin, Holley Pharm, 40 mg dihydroartemisinin/320 mg piperaquine tablets), targeting a total dose of 6.4 and 51.2 mg/kg of dihydroartemisinin and piperaquine, respectively, given in three equally divided daily doses to the nearest quarter tablet. We used a pill cutter to ensure that the tablet fractions were as close to the nearest quarter tablet as possible. Participants in the DP group also received placebo tablets administered in the evening over 3 d to simulate the AL dosing schedule. Study medications were administered with water, and patients were given a glass of milk after each dose of study medication.

All treatment was directly observed. Participants were given the option either to wait at the clinic for the evening dose (lunch was provided) or to leave the clinic and return in the evening (transport was provided). After each dose, children were observed for 30 min, and the dose was readministered if vomiting occurred. All patients were provided with a 3 d supply of acetaminophen for treatment of febrile symptoms. Children with hemoglobin of less than 10 g/dl were treated according to Integrated Management of Childhood Illness guidelines with ferrous sulfate for 14 d and antihelminthic treatment if appropriate. Households of all patients were given two long-lasting insecticide-treated bed nets (ITNs) (PermaNet, Vestergaard Frandsen, http://www.vestergaard-frandsen.com) on the day of enrollment, with instructions for one net to be used by the study patient.

### Objectives

The objectives of the study were to compare the efficacy and safety of AL and DP for the treatment of uncomplicated falciparum malaria at a high transmission–intensity site in Uganda.

### Outcomes: Efficacy

Treatment outcomes were classified according to 2006 World Health Organization (WHO) guidelines as early treatment failure (ETF; danger signs or complicated malaria or failure to adequately respond to therapy days 0–3); late clinical failure (LCF; danger signs or complicated malaria or fever and parasitemia on days 4–42 without previously meeting criteria for ETF or LPF); late parasitological failure (LPF; asymptomatic parasitemia days 7–42 without previously meeting criteria for ETF or LCF); or adequate clinical and parasitological response (absence of parasitemia on day 42 without previously meeting criteria for ETF, LCF, or LPF) [16]. Patients were treated with quinine sulfate (10 mg/kg three times daily for 7 d) on the day that they fulfilled criteria for early treatment failure or late clinical failure. Patients with late parasitological failure were followed, and were given quinine only if they developed fever with parasitemia or remained parasitemic on the last day of follow-up. Patients were excluded from further follow-up after enrollment if any of the following occurred: (1) use of antimalarial drugs outside of the study protocol; (2) withdrawal of consent; (3) loss to follow-up; (4) protocol violation; or (5) death due to a nonmalarial illness.

The primary efficacy outcomes were the 28- and 42-d risks of early treatment failure or recurrent parasitemia (LCF or LPF), unadjusted and adjusted by genotyping. Secondary efficacy outcomes included prevalence of fever and parasitemia during the first 3 d of follow-up, change in mean hemoglobin from day 0 to day 42 or day of repeat therapy, and prevalence of gametocytemia (presence of gametocytes on thick smears) during follow-up in participants lacking gametocytes at enrollment.

Molecular genotyping techniques were used to distinguish recrudescent from new infections for all patients with LCF or LPF response. Briefly, parasite DNA was isolated from filter paper blood samples collected at enrollment and on the day of recurrent parasitemia using chelex extraction. Paired samples were genotyped in a stepwise fashion using *merozoite surface protein (msp)-2, msp-1,* and four microsatellites [17]. If, for any of the six loci, an allele was not shared between day 0 and day of recurrence, the infection was classified as a new infection. If at least one allele was shared between day 0 and day of recurrence at all six loci, the infection was classified as a possible recrudescence. The term “possible recrudescence” was used because the complexity of infection (number of infecting parasite strains) was very high in our setting, making it difficult to distinguish definitively a true recrudescence from a new infection.

### Outcomes: Safety

Secondary safety outcomes included risks of serious adverse events and common adverse events of any severity. An adverse event was defined as any untoward medical occurrence, irrespective of its suspected relationship to the study medications [18]. At each follow-up visit, patients were assessed for any new or worsening event. All events were graded by severity (none, mild, moderate, severe, life-threatening) and relationship to study treatment (none, unlikely, possible, probable, or definite) using guidelines from the World Health Organization (Toxicity Grading Scale for Determining the Severity of Adverse Events) [19]and the United States National Institutes of Health, Division of Microbiology and Infectious Diseases (Pediatric Toxicity Tables, May 2001) [20]. A serious adverse event was defined as any adverse experience that resulted in death, life-threatening experience, inpatient hospitalization, persistent or significant disability or incapacity, or specific medical or surgical intervention to prevent serious outcome.

### Sample Size

We calculated sample size to test the hypothesis that the risk of recurrent parasitemia after 42 d would differ between the two treatment groups. Based on previous data, the risk of recurrent parasitemia (unadjusted by genotyping) after 42 d was estimated to be 50% after treatment with AL [3]. Using this estimate, we calculated that 200 patients (allowing for 10% loss to follow-up) would need to be enrolled in each treatment arm to detect a 15% risk difference between the treatment groups with a two-sided type I error of 0.05 and power of 80%.

### Randomization: Sequence Generation, Allocation Concealment, Implementation

A randomization list was computer generated by an off-site investigator without the use of blocking or stratification. Sequentially numbered, sealed envelopes containing the treatment group assignments were prepared from the randomization list. The study doctors assigned treatment numbers sequentially and the study nurse allocated treatment by opening the envelope corresponding to the treatment number. The randomization codes were secured in a locked cabinet accessible only by the study nurse. Participants were enrolled by the study physicians, and treatments were assigned and administered by the study nurse.

### Blinding

Only the study nurse was aware of treatment assignments. All other study personnel, including the study physicians and laboratory personnel involved in assessing outcomes, were blinded to the treatment assignments. Patients were not informed of their treatment regimen, but the color of the two study medications was not the same (DP and placebo tablets were light blue; AL tablets were light yellow).

### Statistical Methods

Data were entered and verified using Epi Info version 6.04 and analyzed using STATA version 8.0 (STATA, http://www.stata.com). Efficacy and safety data were evaluated using a modified intention-to-treat analysis which included all patients who fulfilled enrollment criteria. Patients who were randomized to therapy but not enrolled in the study due to laboratory results available on day 1 were not included in the analysis. Risks of recurrent parasitemia at 28 and 42 d of follow-up (adjusted and unadjusted by genotyping) were estimated using the Kaplan-Meier product limit formula. Data were censored for patients who did not complete follow-up and for new infections when estimating outcomes adjusted by genotyping. Patients with LCF or LPF due to non-falciparum species were censored as non-failures at the time they were classified as LCF or LPF. The *Z*-test was used to compare the Kaplan-Meier estimates of treatment efficacy at fixed points in time between the treatment groups. Confidence intervals around the difference between Kaplan-Meier estimates were calculated using normal approximation and Greenwood's estimates of standard errors. Categorical variables were compared using Chi-squared or Fisher exact test and continuous variables were compared using the independent samples *t*-test. All reported *p*-values are two sided without adjustment for multiple testing and were considered statistically significant if below 0.05.

## RESULTS

### Participant Flow

**Figure 1:**
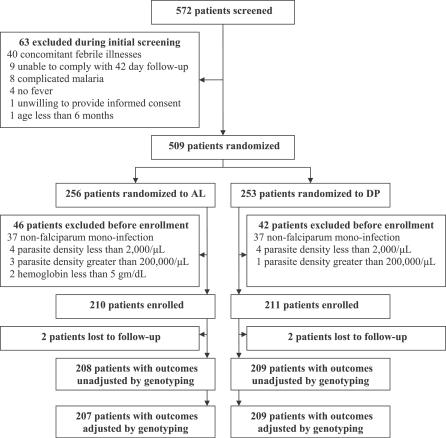
Trial Profile

Of 572 patients screened, 509 were randomized to treatment, and 421 were enrolled in the study (Figure 1). Primary efficacy outcomes, unadjusted and adjusted by genotyping, were available for 417 (99%) and 416 (99%) enrolled participants, respectively. One patient with an unsuccessful genotyping result in the AL group was not included in the analysis when adjusting for genotyping.

### Recruitment

The study was conducted between March and July 2006.

### Baseline Data

Among patients enrolled in the study, there was no difference at baseline of gender, age, temperature, parasite density, hemoglobin, or recent antimalarial use between the two treatment groups (Table 1). Among patients treated with AL, the mean total doses (standard deviation [SD]) were 13.1 (2.8) mg/kg for artemether and 78.8 (17.1) mg/kg for lumefantrine. Among patients treated with DP, the mean total doses (SD) were 7.3 (1.0) mg/kg for dihydroartemisinin and 58.4 (8.0) mg/kg for piperaquine.

**Table 1:**
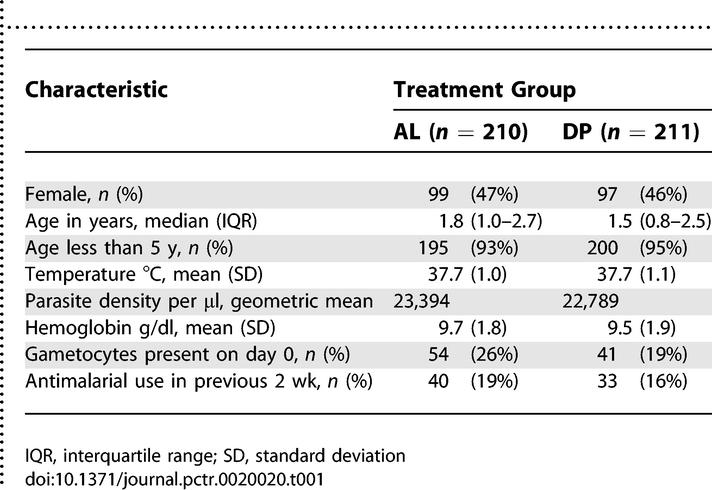
Baseline Characteristics of Participants According to Treatment Group

### Outcomes and Estimation

#### Primary efficacy outcomes.

**Figure 2:**
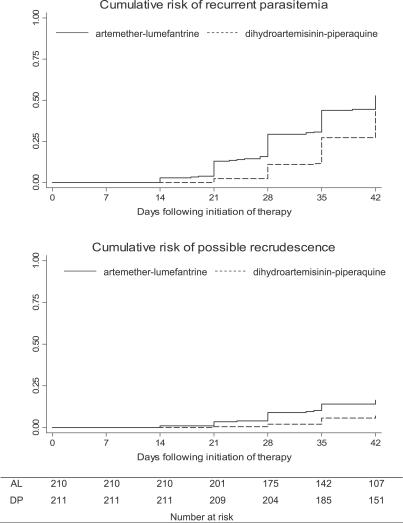
Cumulative Risk of Recurrent Parasitemia Stratified by Treatment Group

There were no early treatment failures in the first 3 d following initiation of therapy. Episodes of recurrent parasitemia were first detected 14 d following therapy in the AL arm and 21 d following therapy in the DP arm (Figure 2; Table 2). The risk of recurrent falciparum parasitemia unadjusted by genotyping was significantly lower for participants treated with DP than for those treated with AL after 28 d of follow-up (11% versus 29%; risk difference [RD] = 18%, 95% confidence interval [CI] 11%–26%) and after 42 d of follow up (43% versus 53%; RD = 9.6%, 95% CI 0%–19%) (Table 3). Similar trends were seen when results were adjusted by genotyping. The risk of recurrent parasitemia due to possible recrudescence was significantly lower for participants treated with DP than for those treated with AL after 28 d of follow-up (1.9% versus 8.9%; RD = 7.0%, 95% CI 2.5%–12%) and after 42 d of follow up (6.9% versus 16%; RD = 9.5%, 95% CI 2.8%–16%) (Table 3). Among patients with recurrent parasitemia, the median time to recurrent parasitemia was significantly shorter in patients treated with AL compared to patients treated with DP (28 d versus 35 d, *p* < 0.0001). Additionally, patients treated with AL had a higher risk of recurrent parasitemia due to non-falciparum species (all were either P. malariae or P. ovale) compared to patients treated with DP (5.2% versus 0.9%, *p* = 0.01). Results were similar when restricting the analyses to children under the age of 5 y, as 94% of patients enrolled were in this age range.

**Table 2:**
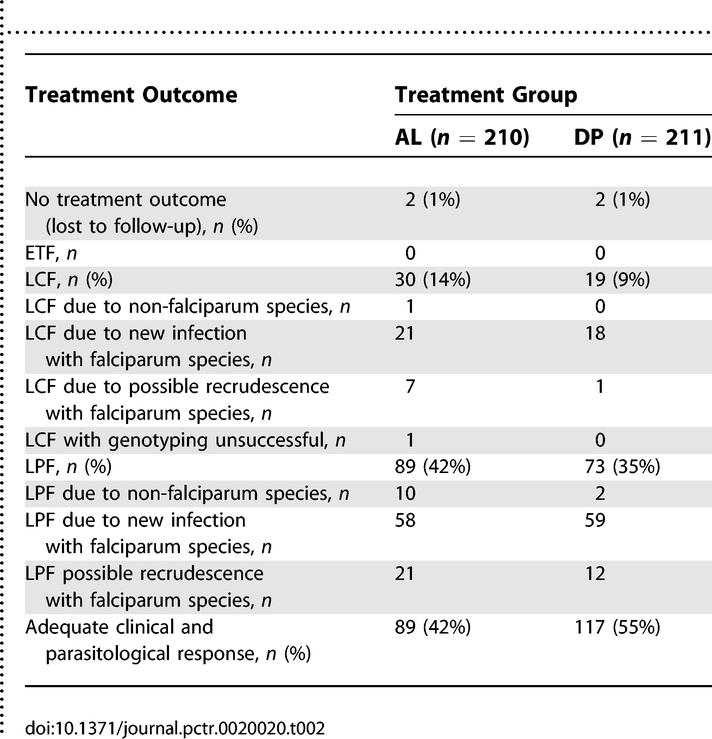
WHO Treatment Outcomes after 42 Days of Follow-Up

**Table 3:**
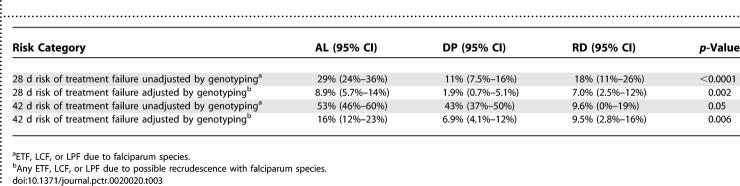
Estimates of Comparative Efficacy

Patients with asymptomatic recurrent parasitemia (LPF) were not treated unless they developed symptomatic malaria or reached the end of the 42-d follow-up period. Among 121 patients with LPF occurring before day 42, only six (5%) spontaneously cleared their parasites without treatment, 86 (53/75, 71% in AL group and 33/46, 72% in DP group) went on to develop symptomatic malaria by day 42, 28 (23%) had persistent asymptomatic parasitemia at day 42, and one (1%) took other antimalarials prior to day 42. Overall, 81 (39%) of 210 patients treated with AL went on to develop recurrent symptomatic malaria, compared to 52 (25%) of 211 patients treated with DP (*p* = 0.002).

#### Secondary efficacy outcomes.

The prevalence of fever (either subjective or documented) was similar over the first 3 d of follow-up in the two treatment groups. Both treatments produced rapid clearance of parasitemia with no parasites detected by day 3 (Table 4). The appearance of gametocytes not present at enrollment was significantly lower over the last 4 wk of follow-up in DP group (Table 4). Patients treated with DP had a higher mean increase in hemoglobin levels, which was of borderline statistical significance (1.9 versus 1.5 g/dl, *p* = 0.05). However, among patients with recurrent parasitemia there was no difference in the prevalence of anemia (Hb <10 g/dl) on the day of failure in the AL group (33/117, 28%) compared to the DP group (25/92, 27%) (*p* = 0.87).

**Table 4:**
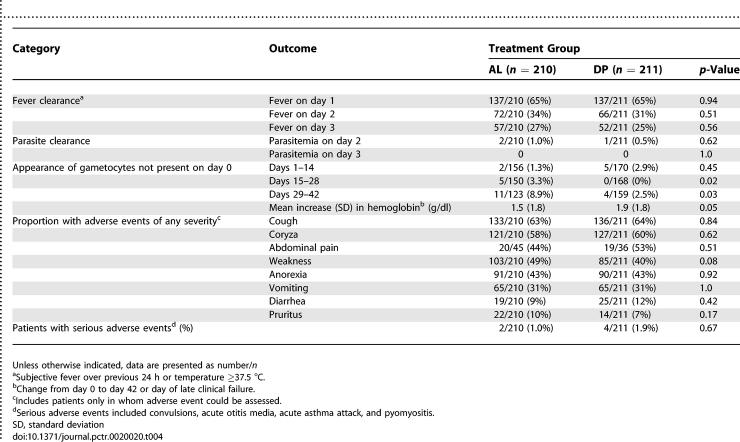
Secondary Outcomes

### Adverse Events

Both drugs were well tolerated. Most adverse events were of mild or moderate severity and consistent with symptoms due to malaria. Overall, there was no difference in the proportion of study participants who experienced any adverse event of moderate or greater severity between the DP (46%) and AL (42%) treatment groups (*p* = 0.47). There was also no difference in the proportion of patients who experienced common adverse events of any severity (Table 4). Serious adverse events occurred in six participants and included three febrile convulsions, one case of acute otitis media, one acute asthma attack, and one case of pyomyositis. All serious adverse events were judged to be unrelated to study medications. No patients were withdrawn from the trial for drug-induced vomiting that would have required alternative treatment.

## DISCUSSION

### Interpretation

In this randomized clinical trial, AL and DP were both highly efficacious at initial clearance of parasitemia and well tolerated for treatment of uncomplicated malaria in Apac, Uganda, an area with extremely high malaria transmission intensity. Importantly, DP-treated patients had a significantly lower risk of recurrent parasitemia in both falciparum and non-falciparum infections. Accurate distinction between recrudescent and new falciparum infections following therapy was challenging due to the complexity of infection in this high transmission setting, despite the use of 6-locus genotyping. However, DP clearly offered better post-treatment prophylactic effect following therapy compared to AL and our data suggest a reduced risk of treatment failure due to recrudescent parasites. DP also offered other benefits, including a lower risk of gametocytemia after therapy and better hemoglobin recovery.

The significantly lower risk of recurrent parasitemia after treatment with DP compared to AL is likely explained by differences in the terminal elimination half-lives of the two partner drugs. Piperaquine, a bisquinoline, is estimated to have an elimination half-life of 2–3 wk [21] compared to lumefantrine, a quinoline, which has an estimated elimination half-life of 4–10 d [22]. Selecting the ideal partner drug to combine with artemisinins in ACT regimens remains a challenge. Extended elimination half-lives may provide better post-treatment prophylaxis, but may also increase the risk for the selection of drug-resistant parasites, especially in areas of intense malaria transmission [23]. Resistance may develop to partner drugs during the elimination phase of the drug (due to their longer half-life), when newly infecting parasites are exposed to subtherapeutic levels of the drug. As ACT use becomes widespread in areas with high levels of malaria transmission, it will be important to monitor closely for the selection of parasites that are resistant to artemisinin partner drugs, since the benefits of a regimen that offers decreased recurrent infection must be balanced with the consequences of increased selection of resistant parasites.

### Generalizability

Our study was conducted in an area of very high malaria transmission and highlights the importance of level of transmission in determining the overall efficacy of an antimalarial treatment regimen [24]. In a high-transmission setting, differences in the post-treatment prophylactic effect of ACTs may have a significant impact on the timing and frequency of recurrent episodes of malaria, as seen in this study. In areas with lower transmission intensity, a drug's post-treatment prophylactic effect would be expected to be of lesser importance. This is illustrated in a study from three sites in Rwanda with differing transmission intensity [14]. At a periurban site with relatively low transmission intensity, the risk of recurrent parasitemia after 28 d was equally low in patients treated with DP (4%) and patients treated with AQ + AS (7%). In contrast, at two rural sites with high transmission intensity, the risk of recurrent parasitemia was significantly lower with DP (12%) compared to AQ + AS (23%).

One limitation of this study was the difficulty of accurately distinguishing recrudescence from new infections among patients with recurrent parasitemia, due to the high complexity of infection. Among episodes classified as “possible recrudescence,” the mean complexity of infection was over five clones on day 0 and approximately four clones on the day of recurrent parasitemia. Even using six-locus genotyping, the probability of a new infection being misclassified as a recrudescence may be relatively high. Thus, the reported 42-d risks of recurrent parasitemia adjusted by genotyping of 16% in the AL treatment arm and 6.9% in the DP treatment arm likely overestimate the true risks of recrudescence.

In this study we followed the new WHO outcome classification system where a patient is classified as a failure after the first reappearance of parasitemia, regardless of whether the patient is symptomatic [16]. Previously, patients with asymptomatic parasitemia following therapy were distinguished from those with recurrent symptomatic malaria based on the assumption that asymptomatic parasitemia may not be clinically important in areas of high transmission intensity [25]. However, in this study, where semi-immunity is expected to be high, the vast majority of patients with asymptomatic parasitemia following therapy went on to develop symptomatic malaria, strongly supporting the new WHO protocol recommendations and suggesting the weakness of natural immunity in clearing these parasites.

### Overall Evidence

In this study, DP was shown to offer benefits over AL, including lower risks of recurrent parasitemia and gametocytemia following therapy and improved hemoglobin recovery. Both DP and AL are fixed-dose coformulated ACTs. However, DP has a simpler, once-daily dosing schedule compared to AL, which is provided twice daily, ideally with a fatty meal. Our results could have important policy implications. Currently AL is the recommended first-line therapy for treatment of uncomplicated malaria in Uganda, with AQ + AS recommended as an alternative if AL is not available. DP is now registered for use in Uganda, and appears to offer an additional highly efficacious ACT for our limited antimalarial armamentarium. In studies from Southeast Asia, DP appears to be well tolerated and highly efficacious against multidrug-resistant falciparum malaria [7–11]. In the only published study evaluating DP in Africa, DP had a cure rate of over 95% and had a significantly lower risk of recurrent parasitemia and adverse events compared to AQ + AS and AQ + SP in Rwanda [14]. Based on available data, DP warrants serious consideration as a first-line therapy for uncomplicated malaria in Africa. DP may also have a role for presumptive treatment of fever through the program for home-based management of fever (HBMF). Currently prepackaged CQ + SP (Homapak) is being distributed in the HBMF program. However, the CQ + SP combination is no longer efficacious [24], and there are plans to incorporate ACTs in the future. Use of DP for HBMF might be attractive because of its simple dosing schedule. There are also potential advantages of having more than one first-line therapy, one for facility-based management of malaria and another for HBMF, in reducing the selective pressure of using one ACT.

Despite the excellent initial parasite clearance of both ACT regimens in this study and the provision of ITNs at enrollment, approximately half of all participants experienced recurrent parasitemia within 42 d. This finding emphasizes the need for more aggressive approaches to malaria control in areas with very high malaria transmission. A study done in an area of South Africa with lower-intensity transmission found that the combination of vector control measures including indoor residual spraying and provision of AL dramatically decreased the malaria burden [26]. In order to reduce new malaria infections in our study population, we anticipate that combining several malaria control measures, including treatment with ACTs, provision of ITNs (with education about their use), and potential use of indoor residual spraying, as in South Africa, will likely decrease the malaria burden and reduce drug pressure due to repeated use of ACTs. Monitoring of the impact of these combined control measures will be critical to assess our success in malaria control in Uganda.

## SUPPORTING INFORMATION

CONSORT Checklist AL versus DP Trial, Apac Uganda (49 KB DOC)

Trial Protocol Comparison of AL and DP for Treatment of Uncomplicated Malaria in Uganda: Evaluation of Efficacy, Safety, and Tolerability at Three Sites with Varying Transmission Intensity (1.1 MB DOC)

## Abbreviations

ACT: artemisinin-based combination therapy
AL: artemether-lumefantrine
AQ: amodiaquine
AS: artesunate
CI: confidence interval
CQ: chloroquine
DP: dihydroartemisinin-piperaquine
ETF: early treatment failure
HBMF: home-based management of fever
ITN: insecticide-treated bed net
LCF: late clinical failure
LPF: late parasitological failure
RD: risk difference
SD: standard deviation
SP: sulfadoxine-pyrimethamine
WBC: white blood cell
WHO: World Health Organization
